# Prognosis of three histological subtypes of colorectal adenocarcinoma: A retrospective analysis of 8005 Chinese patients

**DOI:** 10.1002/cam4.2234

**Published:** 2019-05-10

**Authors:** Chao Li, Hongtu Zheng, Huixun Jia, Dan Huang, Weilie Gu, Sanjun Cai, Ji Zhu

**Affiliations:** ^1^ Department of Radiation Oncology Fudan University Shanghai Cancer Center Shanghai China; ^2^ Department of Oncology Shanghai Medical College, Fudan University Shanghai China; ^3^ Department of Colorectal Surgery Fudan University Shanghai Cancer Center Shanghai China; ^4^ Shanghai general hospital Shanghai China; ^5^ Department of Pathology Fudan University Shanghai Cancer Center Shanghai China

**Keywords:** colorectal adenocarcinoma, mucinous, prognosis, signet ring, stage

## Abstract

**Background and objectives:**

To evaluate the effect of the varied histological subtypes on clinical outcomes and to determine the prognostic implications of mucinous adenocarcinomas (MAC) and signet ring cell carcinomas (SRCC) compared with classic adenocarcinomas (AC).

**Methods:**

A total of 8005 patients, including 7502 AC, 428 MAC and 75 SRCC, who underwent definitive surgery between 2007 and 2015 at Fudan University Shanghai Cancer Center were remained for analysis in this study.

**Results:**

MAC and SRCC were more common in right‐sided colon cancer, in males and in young patients, compared to AC; moreover, MAC and SRCC led to a higher probability to develop lymph node metastasis, lymphovascular invasion and perineural invasion. For survival outcomes, we found that the 5‐year overall survival (OS) of SRCC was significantly lower than that of MAC and AC, while the 5‐year OS of MAC is much lower than that of AC. However, in multivariable analysis, the difference in survival between SRCC, MAC and AC was no longer significant, especially when stratified by N stage.

**Conclusions:**

MAC and SRCC are rare subtypes of colorectal cancer with a higher T stage, N stage as well as higher incidence of lymphovascular and nerve invasion. However, neither MAC nor SRCC was an independent predictor of decreased survival in multivariate analysis.

## 1 INTRODUCTION

Colorectal cancer (CRC) is one of the leading causes of cancer mortality worldwide.^1^ CRC is the third most common malignancy in European and American countries,^1^, ^2^ while in China, incidence and mortality rates have been increasing continuously in both colon and rectal cancer because of diet change and aging population.^3^ More than 90% of colorectal carcinomas are adenocarcinomas derived from epithelial cells of the colorectal mucosa.^4^ And the World Health Organization (WHO) pathologic classification of gastrointestinal lists a quantity of histologic subtypes of colorectal carcinomas, such as classic adenocarcinomas (AC), mucinous adenocarcinomas (MAC), signet ring cell carcinomas (SRCC) and other rare variants of colorectal carcinomas including squamous cell, neuroendocrine, adenosquamous, spindle cell, and undifferentiated carcinomas.^4^, ^5^ Apart from TNM staging system, histologic classification of CRC may influence the clinical features and outcome, thus, to clarify the effect of the varied histological subtypes will help clinicians choose the appropriate treatment strategy.

It is reported previously that approximately 10% of all CRC are MAC, and approximately 1% are SRCC.^6^, ^7^, ^8^ Because of the relatively rare incidence, the evaluation of clinicopathologic characteristics and survival of MAC and SRCC are difficult, recent studies suggest that MAC and SRCC were histological variants of CRC with different characteristics than AC, such as more advanced tumor stage at presentation, a younger patient age, more female patients and more distinct molecular features.^8^, ^9^ Additionally, it has been considered that SRCC and MAC carries a worse prognosis than AC.^10^ especially SRCC. However, the small sample size of these studies, which reduced the chance of detecting a true effect, may also cause unreliable conclusion.

The purpose of the study was to evaluate the effect of varied histological subtypes on clinical outcome and to determine the prognostic implications of MAC and SRCC compared with AC.

## 2 MATERIALS AND METHODS

### 2.1 Patient selection

This study was designed as a retrospective investigation of data collected for all patients who underwent definitive surgery without neoadjuvant chemoradiation or chemotherapy at Fudan University Shanghai Cancer Center (FDUCC) from 2007 to 2015. The inclusion criteria for this study were as follows: (a) history of primary colorectal cancer; (b) histologically confirmed adenocarcinoma (including AC, MAC and SRCC); (c) undergoing definitive surgery; (d) No evidence of distant metastasis at initial presentation; (e) no history of neoadjuvant therapy because neoadjuvant therapy caused tumor regression led to inaccurate staging. All data were collected from the database of FDSCC, including demographic features, preoperative tumor staging, details of the surgical procedure, postoperative histopathology, and follow‐up (date of last visit, date and site of tumor recurrence, date of death). Rectal cancer is defined as cancer located within 12 cm of the anal verge in our center and the colon cancer was divided into right‐side (including cecum, ascending colon, and right half of transverse colon) and left‐side (including left half of transverse colon, descending colon, and sigmoid colon) according to National Comprehensive Cancer Network guidelines. For each patient, the evaluation included a complete history and physical examination, including digital examination, complete blood count, hepatic and renal function tests, tumor marker measurement, colonoscopy and biopsy, computed tomography (CT) of the thorax and abdomen, magnetic resonance imaging (MRI) of the pelvis, and in selected patients, endorectal ultrasound and PET. Patient follow‐up was scheduled every three months during the first two years, and then every six months over the next three years. After five years, the frequency of follow‐up was extended to once each year. All patients were restaged according to the 7th edition of the TNM system from the American Joint Committee on Cancer (AJCC). The study was approved by the Institutional Review Board of the Fudan University Shanghai Cancer center and conducted in accordance with the Declaration of Helsinki of 1975 in its most recent version. Patients provided written informed consent prior to the study.

### 2.2 Pathological examination

The CRC of all patients was classified as AC, MAC, or SRCC according to the WHO pathologic classification of gastrointestinal tumors.^5^ MAC is defined by >50% of the tumor volume composed of extracellular mucin, typically showing large glandular structures with pools of extracellular mucin. The presence of >50% of tumor cells showing signet ring cell features characterized by a prominent intracytoplasmic mucin vacuole that pushes the nucleus to the periphery is classified as SRCC. AC is defined by classical glandular formation and configuration of the glandular structures.

### 2.3 Statistical analysis

The overall survival (OS) and the disease‐free survival (DFS) rates were estimated by using the Kaplan‐Meier method, and comparison of three groups was applied by the log‐rank test. Cox proportional hazards regression was used for univariate and multivariate modeling and for examining the prognostic significance of the variables identified in the models. A *P*‐value of <0.05 was considered statistically significant.

## 3 RESULTS

### 3.1 Demographic and clinicopathologic characteristics

A total of 9015 patients with colorectal adenocarcinoma undergoing definitive surgery between 2007 and 2015 at FUSCC were enrolled in this study. However, 1010 of these patients were excluded because of neoadjuvant therapy. Therefore, 8005 cases remained for analysis, including 428 MAC and 75 SRCC. The demographic and clinicopathologic characteristics are listed in Table 1. Median follow‐up duration are 23.8, 26.9, and 26.9 months for AC, MAC, and SRCC groups, respectively.

**Table 1:** Baseline clinicopathologic parameters

			Tumor subtype			Total	*P* value
			AC	MAC	SRCC		
Patients (n)			7502 (93.7%)	428 (5.3%)	75 (0.9%)	8005	
Sex (n)	Male	Num	4429	235	54.0	4718.0	
		%	59.0%	54.9%	72.0%	58.9%	
	Female	Num	3073	193	21.0	3287	
		%	41.0%	45.1%	28.0%	41.1%	0.017
Age (year)	<=35	Num	224	33	14	271	
		%	3.0%	7.7%	18.7%	3.4%	
	35‐55	Num	2312	158	33	2503	
		%	30.8%	36.9%	44.0%	31.3%	
	55‐75	Num	4365	206	26	4597	
		%	58.2%	48.1%	34.7%	57.4%	
	>75	Num	601	31	2	634	
		%	8.0%	7.2%	2.7%	7.9%	<0.001
Location	Rectum	Num	4188	182	38	4408	
		%	55.8%	42.5%	50.7%	55.1%	
	Left hemicolon	Num	1606	80	8	1694	
		%	21.4%	18.7%	10.7%	21.2%	
	Right hemicolon	Num	1674	164	29	1867	
		%	22.3%	38.3%	38.7%	23.3%	
	Missing	Num	34	2	0	36	
		%	0.5%	0.5%	0.0%	0.4%	<0.001
T Stage	T1	Num	457	4	1	462	
		%	6.1%	0.9%	1.3%	5.8%	
	T2	Num	1370	39	1	1410	
		%	18.3%	9.1%	1.3%	17.6%	
	T3	Num	2714	174	36	2924	
		%	36.2%	40.7%	48.0%	36.5%	
	T4	Num	2926	208	37	3171	
		%	39.0%	48.6%	49.3%	39.6%	
	Missing	Num	35	3	0	38	
		%	0.5%	0.7%	0.0%	0.5%	<0.001
N Stage	N0	Num	3945	187	11	4143	
		%	52.6%	43.7%	14.7%	51.8%	
	N1	Num	2050	96	12	2158	
		%	27.3%	22.4%	16.0%	27.0%	
	N2	Num	1507	145	52	1704	
		%	20.1%	33.9%	69.3%	21.3%	<0.001
lymphovascular invasion	‐	Num	5603	311	22	5936	
		%	74.7%	72.7%	29.3%	74.2%	
	+	Num	1811	110	52	1973	
		%	24.1%	25.7%	69.3%	24.6%	
	Missing	Num	88	7	1	96	
		%	1.2%	1.6%	1.3%	1.2%	<0.001
Nerve invasion	‐	Num	5915	340	20	6275	
		%	78.8%	79.4%	26.7%	78.4%	
	+	Num	1533	85	55	1673	
		%	20.4%	19.9%	73.3%	20.9%	
	Missing	Num	54	3	0	57	
		%	0.7%	0.7%	0.0%	0.7%	<0.001
CRM	‐	Num	6888	365	63	7316	
		%	91.8%	85.3%	84.0%	91.4%	
	+	Num	84	21	9	114	
		%	1.1%	4.9%	12.0%	1.4%	
	Missing	Num	530	42	3	575	
		%	7.1%	9.8%	4.0%	7.2%	<0.001

Abbreviation: CRM: Circumferential resection margin

Patients with SRCC were younger than those with MAC or AC. There were significant differences in age, such that 18.7% of the patients with SRCC were <35 years old compared with 7.7% and 3.0% of those with MAC and AC, respectively (*P* < 0.001). Additionally, there was a much higher proportion of male in patients with SRCC (72.0%, *P* = 0.034) than the proportions for AC (59.0%) and MAC (54.9%). Compared with AC (22.3%), MAC (38.3%) and SRCC (38.7%, *P* < 0.001) were located more frequently in the right hemicolon. Furthermore, SRCC and MAC were diagnosed at more advanced stages than AC (*P* < 0.001 in both T and N stage); in particular, 63.9% of patients with SRCC were with N2 stage compared with AC (20.1%) and MAC (33.9%). The positive CRM status was observed in 1.6% (66/4109) and 1.4% (48/3321) in rectum and colon. Circumferential resection margin (CRM) positive was showed significantly more frequently for patients with SRCC than for those with AC and MAC (12% vs 4.9% vs 1.1%, *P* < 0.001). Similarly, both lymphovascular invasion (69.3% vs 24.1% vs 25.7%, *P* < 0.001) and nerve invasion (73.3% vs 19.9% vs 20.4%, *P* < 0.001) occurred more frequently in patients with SRCC than those with AC and MAC.

### 3.2 Survival outcomes

**Figure 1:**
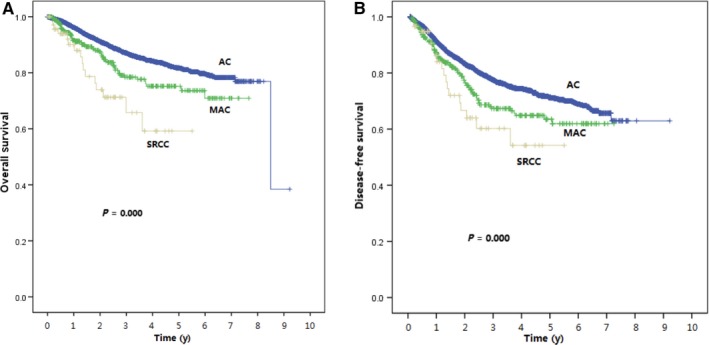
Kaplan‐Meier Curve Plotting the 5‐year OS (A) and DFS (B) Corresponding to the Three Different Histologic Subtypes

The 5‐year DFS and 5‐year OS were significantly lower for the SRCC than AC and MAC (*P* < 0.001). The 5‐year OS for the patients with SRCC was 64.2% whereas those with AC and MAC were 82.0% and 64.2% (*P* < 0.001). Similarly, the 5‐year DFS was 71.6% for patients with AC, 64.3% for patients with MAC, and 54.4% for patients with SRCC (*P* < 0.001). The Kaplan‐Meier survival curves for different histological subtypes are displayed in Figure 1.

In addition to the histological subtypes of cancer (AC, MAC, or SRCC), factors associated with survival on univariate analysis were age, T stage, N stage, lymphovascular invasion, nerve invasion, CRM, and tumor location. Most of these factors remained independent prognostic factors except tumor location in the multivariate Cox model (Table 2). However, there were no significant differences in OS and DFS between SRCC, MAC and AC after multivariate Cox regression analysis, no matter AC was compared with SRCC or MAC.

**Table 2:** Independent prognostic factors in multivariable analysis of overall survival and disease‐free survival

	OS			DFS		
	*P* value	HR	95% CI for HR	*P* value	HR	95% CI for HR
Age	<0.001			<0.001		
35‐55 vs <35	0.043	1.321	0.801‐2.180	0.107	1.153	0.803‐1.656
55‐75 vs <35	0.001	1.724	1.053‐2.821	0.003	1.423	0.997‐2.031
>=75 vs <35	<0.001	3.990	2.357‐6.756	<0.001	1.886	1.262‐2.817
T stage	<0.001			<0.001		
T2 vs T1	0.161	1.810	0.769‐4.259	0.161	1.487	0.842‐2.627
T3 vs T1	0.037	2.382	1.045‐5.432	0.004	2.208	1.282‐3.805
T4 vs T1	0.001	4.109	1.816‐9.295	<0.001	3.141	1.831‐5.388
Vascular thrombosis						
(+) vs (‐)	<0.001	1.592	1.328‐1.910	<0.001	1.419	1.225‐1.643
Nerve invasion						
(+) vs (‐)	0.013	1.264	1.057‐1.513	<0.001	1.599	1.386‐1.843
CRM						
(+) vs (‐)	<0.001	2.495	1.781‐3.495	0.014	1.515	1.095‐2.097
N stage	<0.001			<0.001		
N1 vs N0	<0.001	1.608	1.271‐2.036	<0.001	1.464	1.271‐2.036
N2 vs N0	<0.001	3.408	2.697‐4.308	<0.001	2.925	2.436‐3.513
Tumor subtype	0.690			0.307		
MAC vs AC	0.459	1.112	0.822‐1.505	0.915	1.018	0.790‐1.311
SRCC vs AC	0.628	1.173	0.669‐2.059	0.128	0.678	0.403‐1.140
Location	0.003			0.119		
Left hemicolon vs rectum	0.596	0.946	0.765‐1.169	0.437	0.942	0.798‐1.111
Right hemicolon vs rectum	0.003	1.308	1.082‐1.580	0.118	1.130	0.967‐1.320

### 3.3 N stage‐stratified survival outcomes of SRCC

**Figure 2:**
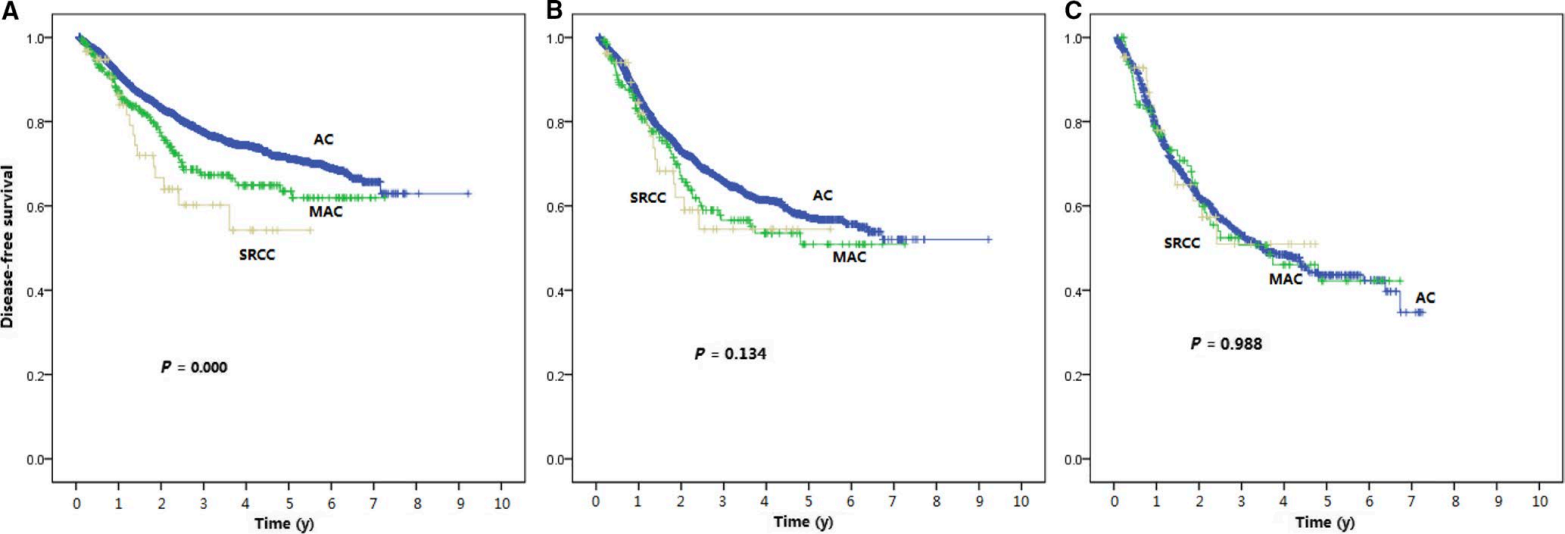
DFS for Patients in All (A)/N+ (B)/N2 (C) Group, Corresponding to Adenocarcinoma, Mucinous Adenocarcinoma, and Signet Ring Cell Carcinoma. Levels of significance are listed in Table 3

We performed an N stage‐stratified survival analysis to evaluate the survival outcomes more precisely. Our data suggested that when analyzed based on node positive group, there were no significant differences of 3y‐DFS and 5y‐DFS between AC, MAC, and SRCC, although patients with SRCC and MAC performed a trend toward shorter survival than patients with AC (3y‐DFS: 54.9% vs 57.10% vs 66.2%, *P* = 0.136, Table 3). Furthermore, AC, MAC and SRCC had almost the same 3y‐DFS stratified by N2 stage (53.3% vs 51.7% vs 51.9%, *P* = 0.996), and the same result showed in 5y‐DFS (43.8% vs 43.8% vs 51.9%, *P* = 0.996). (Table 3 and Figure 2).

**Table 3:** DFS for patients in All/N+/N2 group, corresponding to adenocarcinoma, mucinous adenocarcinoma, and signet ring cell carcinoma

	All Group			N+			N2		
	3y	5y	*P*	3y	5y	*P*	3y	5y	*P*
AC	77.80%	71.60%		66.20%	57.80%		53.30%	43.80%	
MAC	67.70%	64.30%		57.10%	51.80%		51.70%	43.80%	
SRCC	60.50%	54.40%	<0.001	54.90%	54.90%	0.136	51.90%	51.90%	0.996

## 4 DISCUSSION

To our knowledge, this study was reported as the largest Chinese cohort investigating patients with AC, MAC, and SRCC and analyzed the relationship between pathologic types and clinical features as well as survival outcomes in 8005 patients with colorectal cancer under a single‐center database. We found that SRCC and MAC were more common in male patients and more likely to lead to lymph node metastasis, lymphovascular invasion, and perineural invasion. We also found that both MAC and SRCC, which presented at a more advanced T stage compared with AC, occurred more often in young patients and right‐sided colon cancer. In addition, CRM positivity was higher in MAC and SRCC compared with AC. For survival outcomes, we found that the 5‐year OS of SRCC was significantly lower than that of MAC and AC, while the 5‐year OS of MAC was much lower than AC. However, neither MAC nor SRCC was an independent predictor of decreased survival on multivariate.

Among 8005 patients recruited in our study, rates of SRCC, MAC, and AC were 0.9%, 5.3%, and 93.7%, respectively, which were consistent with population‐based data sets reported in the Western countries and Asia.^10^ An analysis of 164,628 CRC patients enrolled from 1991 to 2000 based on SEER database in US found that the rates of SRCC, MAC, and AC were 0.9%, 10.3%, and 88.8%, respectively.^1^ Another analysis from the National Cancer Data Base, which included 244,794 CRC patients, reported that SRCC accounted for 0.9%, MAC accounted for 10.4%, and AC accounted for 88.6% of all CRC histologic types.^10^ We could draw a conclusion from the data above that the proportion of MAC is five to ten times more than SRCC but is still much less than AC. Another analysis based on 273 patients with rectal cancer in India reported that proportion of SRCC was 13.6%, whereas the proportion of MAC was only 7.7%.^11^ The inconsistency between publications implies the proportion of SRCC and MAC may associate with different races and tumor sites.

We found that the proportion of male patients in SRCC was 72.0%, which was significantly higher than the proportion of females. This conclusion was consistent with the study by Wei et al^12^ who analyzed 61 CRC patients with SRCC from China. Other analyses performed in the United States,^9^, ^10^ India,^11^ and Singapore^13^ reported similar proportions for gender SRCC and MAC. However, another report by Tan et al^14^ which analyzed 2454 cases of Chinese CRC patients, did not find any difference between proportion of male and female patients in SRCC or MAC histology.

In addition to the features mentioned above, we found that MAC and SRCC more frequently occurred in right‐sided colon compared with AC. This finding is consistent with previous reports.^10^14 However, an analysis based on SEER dataset in the US reported that MAC was more common in the left side of the colon, and another report in 2016 focusing on Chinese patients reported that SRCC more often presented in the left side of the colon and in the rectum.^15^ Prevalence of differences between right‐sided and left‐sided colon cancer was a prominent topic at the 2016 ASCO meeting,^16^ and it was reported that the activation of MSI, which was a major reason of hereditary CRC such as Lynch syndrome,^17^ occurred more frequently in right‐sided colon. MAC had been reported to more frequently show MSI phenotype.^18^, ^19^, ^20^ Recently, Shen et al^12^ divided SRCC into two groups according to the percentage of signet ring cells in tumors >50% and <50% and analyzed 13 genes associated with hereditary tumor syndrome, including MLH1, MSH2, MSH6, and PMS2, and found no difference of pathological mutation between two groups. However, 79.6% of SRCC carried at least one pathogenic mutation. Therefore, it warrants further study whether different histology results in the different molecular features between right‐sided and left‐sided colon cancer.

In accordance with the majority of published studies before,^10^, ^11^, ^21^, ^22^, ^23^ SRCC was more common in young patients and associated with higher T stage, higher N stage, lymphovascular invasion and perineural invasion. This result suggested that SRCC had a stronger tendency of invasion and metastasis compared with AC, and patients with SRCC may already have subclinical metastases even before undergoing radical surgery. CRM positivity was much higher in SRCC than MAC or AC, in our study, as the rate of CRM positivity in SRCC, MAC, and AC was 12.0%, 4.9%, and 1.1%, respectively. Another analysis which focused on rectal cancer in India reported a positive CRM rate of 13.6% in SRCC, which was also significantly higher than AC.^11^ The reason of higher CRM positivity of SRCC may be explained by the fact that SRCC presented with later T stage more frequently. Since CRM positivity means that the patient does not receive R0 surgery and emerges as a significantly negative prognostic factor, patients with SRCC may require a more extensive resection compared with those with AC, and intraoperative frozen sections should be used if necessary. For MAC, its clinical features were between AC and SRCC.

Contrary to our expectations, we found that neither SRCC nor MAC was an independent prognostic factor for DFS and OS in patients with CRC after adjustment for other risk factors. The prognostic significance of SRCC and MAC in CRC patients has long been controversial. In some studies, SRCC and MAC were considered to be a negative prognostic factor for CRC.^23^, ^24^, ^25^ An analysis based on NCBD dataset reported that SRCC was an independent negative prognostic factor for colorectal cancer, while MAC was a negative prognostic factor only for rectal cancer, but not for colon cancer.^10^ Another study based on SEER dataset found that MAC was an independent negative prognostic factor for rectal cancer, a protective prognostic factor for right‐sided colon cancer and had no prognostic effect in left‐sided colon cancer.^9^ The results of these two studies based on large population suggested that SRCC and MAC in different primary site may have different prognostic effect on OS of CRC patients. However, an analysis based on a small sample of Indian population showed no significant difference of OS among MAC, SRCC, and AC in rectal cancer.^11^ A study performed in Italy also suggested that there was no significant association of MAC with the prognosis of CRC patients compared with non‐MAC.^26^

Our results suggest that histological subtypes, such as SRCC and MAC, may not be an independent prognostic factor of CRC patients, instead, SRCC and MAC present a more advanced stage to indirectly affect the prognosis of CRC patients, as previously reported.^9^, ^13^, ^26^ To confirm our hypothesis, we performed a subgroup analysis of N stage and found that although DFS of SRCC and MAC was significantly shorter than that of AC in the univariate analysis, there was no significant difference of DFS among SRCC, MAC and AC in patients with positive lymph node metastases, especially in patients with N2 stage. Our findings highlight the importance of early diagnosis and treatment of CRC. If a patient received the resection with an early stage, that was, with no lymph node metastasis, lymphovascular invasion or perineural invasion, the histology itself may not affect the survival outcome of the patient.

There are several limitations. First, the patient data we collected were from a single cancer center which may lead to selection bias. However, the level of pathological diagnosis in our center is of leading in China, and the highly standardized pathological diagnosis minimized the diagnostic bias. Then, the study was not a prospective cohort, which had some of the inherent inadequacies of retrospective investigations. For example, we only evaluated patients undergoing curative surgery, the other patients of colorectal cancer without radical surgery could not be evaluated because of information loss. And because many patients were from other cities and accepted adjuvant chemotherapy in local hospitals after surgery, it was difficult for us to ensure the strict implementation and accurate evaluation of chemotherapy in the follow‐up process. Furthermore, its statistical power was limited due to the rarity of SRCC. However, we believe that the 75 patients of SRCC assure the statistical power quite well.

## 5 CONCLUSION

MAC and SRCC are presented at a higher T stage, N stage as well as higher incidence of lymphovascular invasion and nerve invasion, which usually considered as independent prognostic factors to poor survival. However, our data indicated that when analyzed through multivariable analysis, the difference in survival between SRCC, MAC and AC was no longer significant, especially when stratified by N stage. Therefore, more research, including molecular mechanism and gene expression, is needed to clarify the relationship between SRCC and N stage, which may benefit the precision medical treatment based on histologic type.

## CONFLICT OF INTEREST

The authors report no conflicts of interest in the work.

## AUTHOR CONTRIBUTIONS

Ji Zhu and Sanjun Cai designed the trial; Chao Li and Hongtu Zheng drafted and revised the manuscript; Ji Zhu and Huixun Jia controlled the quality of data and algorithms; Dan Huang and Weilie Gu helped the manuscript preparation; and all authors read and approved the final manuscript.

## Data Availability

The data that support the findings of this study are available on request from the corresponding author. The data are not publicly available due to privacy or ethical restrictions.
